# Alleviating distressing intrusive memories in depression: A comparison between computerised cognitive bias modification and cognitive behavioural education

**DOI:** 10.1016/j.brat.2014.03.001

**Published:** 2014-05

**Authors:** Jill M. Newby, Tamara Lang, Aliza Werner-Seidler, Emily Holmes, Michelle L. Moulds

**Affiliations:** a Clinical Research Unit for Anxiety and Depression (CRUfAD), School of Psychiatry, The University of New South Wales (UNSW) at St Vincent's Hospital, Level 4, The O'Brien Centre St. Vincent's Hospital, 394-404 Victoria Street Darlinghurst, Sydney, NSW 2010, Australia; b School of Psychology, Level 10 Mathews Building, The University of New South Wales (UNSW), Kensington, Sydney, NSW 2052, Australia; c MRC Cognition and Brain Sciences Unit, 15 Chaucer Road, Cambridge CB27EF, United Kingdom; d Sydney Children's Hospital, High Street Randwick, NSW 2031, Australia

**Keywords:** Intrusive memory, Memory, Depression, Thoughts, Cognitive bias modification, Cognitive behaviour therapy, Imagery

## Abstract

Negative appraisals maintain intrusive memories and intrusion-distress in depression, but treatment is underdeveloped. This study compared the efficacy of computerised bias modification positive appraisal training (CBM) versus a therapist-delivered cognitive behavioural therapy session (CB-Education) that both aimed to target and alter negative appraisals of a negative intrusive autobiographical memory.

Dysphoric participants (Mean BDI-II = 27.85; *N* = 60) completed baseline ratings of a negative intrusive memory, negative appraisals and the Impact of Event Scale, and were randomly allocated either one session of CBM, CB-Education, or a no intervention monitoring control condition (Control). Mood and intrusion symptoms were assessed at one week follow-up.

For all groups, there were significant reductions over one week in mood (depression and anxiety), memory intrusiveness and negative appraisals. Groups differed in terms of intrusion-related distress, with the CB-Education group showing greatest reduction, followed by the CBM group.

The study provides evidence for the link between maladaptive appraisals of intrusive memories and distress in depressed mood. Further, both a single session of CB-Education and (to a lesser degree) CBM are useful in reducing intrusion-related distress. This study may have been underpowered to detect differences and replication is needed with larger samples.

## Highlights

• We evaluated techniques that targeted negative appraisals of intrusive memories.
• Compared one-session CBM and CBT interventions, versus Control.
• CBT education was more effective than control in reducing distress at one week.
• CBM of appraisals led to intermediate reductions in distress at one week.
• Maladaptive appraisals of intrusions can be effectively reduced in depression.

## Introduction

Similar to posttraumatic stress disorder (PTSD), depression is characterised by recurrent and distressing negative intrusive memories (Kuyken & Brewin, 1994). These memories are vivid images, highly avoided, impair daily functioning, and perpetuate depression (Brewin, Reynolds, & Tata, 1999; Newby & Moulds, 2011), yet they are a neglected feature of the disorder. In contrast to PTSD in which trauma memories are the core focus of psychological treatment, traditional forms of cognitive behaviour therapies (CBT) for depression do not typically target intrusive memories (Beck, Rush, Shaw, & Emery, 1979). Additionally, despite preliminary evidence for the use of imaginal exposure and imagery rescripting to reduce such memories in depression (e.g., Kandris & Moulds, 2008; Wheatley et al., 2007), few studies have evaluated techniques that aim to target and reduce intrusive memories in depressed samples.

Mirroring PTSD findings, the way an individual appraises the experience of an intrusive memory appears to be critical in depression (Moulds, Kandris, Williams, & Lang, 2008; Newby & Moulds, 2010). Applying cognitive models of PTSD to depression (Ehlers & Clark, 2000), studies have demonstrated that the degree to which an individual assigns negative interpretations/appraisals to their intrusive memories (e.g., “This memory means I am going crazy”), rather than memory frequency, is a key driver of the distress experienced upon remembering the event. Negative appraisals of intrusions are positively correlated with depression symptoms, intrusion-related distress and cognitive avoidance strategies (e.g., Starr & Moulds, 2006; Williams & Moulds, 2008), and have been shown to predict depression symptoms at six month follow-up over and above baseline symptoms, indicating a maladaptive maintaining role (Newby & Moulds, 2011). Together, these findings suggest that it could be clinically useful to directly target maladaptive negative appraisals of intrusive memories in the treatment of depression. Training depressed individuals, either implicitly or explicitly, to reappraise intrusive memories in a more adaptive way (e.g., “intrusive memories are normal”) may be one possible approach by which to facilitate a reduction in intrusion-related distress. The aim of the current study is to investigate this possibility.

One method that has recently shown promise in modifying appraisals of intrusive memories is computerised cognitive bias modification (CBM). In a non-clinical sample, Lang, Moulds, and Holmes (2009) found that non-explicit, systematic computerised training of positive appraisals of image-based intrusions served to protect against the deleterious effects of a depressing film. Compared to participants trained to adopt negative appraisals, those trained to have positive appraisals of intrusions (positive CBM) *prior to* watching a depressing film went on to have fewer intrusive memories of the film, lower Impact of Event Scale scores (IES, a measure of memory intrusiveness and avoidance, Horowitz, Wilner, & Alvarez, 1979) and lower intrusion-related distress at one week. This study was the first to demonstrate that CBM techniques can successfully modify appraisals of intrusions, leading to reductions in important intrusive memory variables. Similarly, Woud et al. showed that systematic computerised training in a positive reappraisal style *following* a distressing film resulted in less frequent intrusive memories of the film and intrusion distress over the subsequent week (Woud, Holmes, Potsma, Dalgleish, & Mackintosh, 2012), relative to participants who received negative reappraisal training (see also Woud, Postma, Holmes, & Mackintosh, 2013).

These studies raise important questions: (1) Can the benefits of positive CBM training be extended beyond memories of a film to intrusive image-laden autobiographical memories (e.g., divorce or bereavement)? (2) Can the results be extended from a healthy sample to dysphoric or depressed participants? (3) Is a single CBM session of positive appraisal training as efficacious in reducing intrusive memories as a traditional therapist-delivered CBT session that explicitly facilitates more adaptive appraisals of intrusive memories?

Traditional cognitive therapy techniques such as cognitive restructuring and behavioural experiments are typically used to modify maladaptive cognitions in depression. In trauma-focused CBT, these techniques are used to change maladaptive appraisals of the trauma and intrusive re-experiencing (e.g., Ehlers, Clark, Hackmann, McManus, & Fennell, 2005). However, it is unknown whether such techniques are useful in targeting maladaptive appraisals of intrusive memories in depression, and whether CBT techniques can also reduce intrusive memory frequency and distress in depression. Therefore, we designed a focused single session of cognitive behavioural education using cognitive therapy techniques (CB-Education) to act as a therapist-delivered control condition for the computerised CBM. Both sessions (CBM and CB-Education) targeted the same specific mechanism: negative appraisals of intrusive memories. Direct comparisons between CBM and CBT can provide further insight into the therapeutic utility of CBM as a stand-alone intervention for changing maladaptive cognitive biases that characterise depression. Bowler and colleagues showed CBM and computerised CBT were equally efficacious in reducing social anxiety symptoms (Bowler et al., 2012). However, no previous studies have directly compared the relative efficacy of a *face-to-face* CBT session and CBM, and none have compared CBM and CBT in depressed samples.

In this study, participants with mild to moderate depressed mood (dysphoria) described a negative intrusive autobiographical memory, and were then randomly assigned to either positive CBM training, a CB-Education session, or a no-intervention monitoring condition to control for non-specific effects of assessment and self-monitoring (Control). Consistent with previous studies, we assessed frequency, distress and IES scores at baseline. Participants completed a daily diary of their intrusive memory for one week, and were followed up one week later to assess their experience of the intrusive memory. We expected that both the active training conditions (CB-Education and CBM) would be more effective than the Control condition at reducing intrusion-related distress, negative appraisals, memory frequency and IES scores over the course of the week. We also predicted CBM and CB-Education would demonstrate comparable efficacy based on the findings from Bowler et al. (2012).

## Method

### Participants

Participants (*N* = 90) were recruited from the community via posters and online advertisements and screened for eligibility for inclusion in the study via email or phone. Twenty two were excluded on the basis of their responses (self-reported bipolar disorder: *n* = 2; not currently depressed: *n* = 5, no negative intrusive memories over the past week: *n* = 3, insufficient English: *n* = 3, not contactable: *n* = 5, unsuitable for other reasons: *n* = 4). Sixty eight were invited to proceed with the study, and a further 8 were excluded at face-to-face interview because they met DSM-IV criteria for PTSD. This left 60 participants who were randomly allocated using alternation to either the CB-Education (*n* = 20), CBM (*n* = 20) or Control (*n* = 20).^1^ Participants were reimbursed ($AUD20/hour), and all had BDI-II scores >12 indicating mild to extremely severe symptoms (Beck, Steer, & Brown, 1996). All participants attended the one-week follow-up appointment.

### Measures

*The Structured Clinical Interview for the DSM-IV Axis I Disorders* (SCID-I, First, Spitzer, Gibbons, & Williams, 1996) assessed current and lifetime DSM-IV diagnoses of Major Depressive Disorder (MDD), Dysthymia (current), PTSD and Acute Stress Disorder (ASD). The SCID-I was administered by fully qualified Masters-level clinical psychologists (JN, AW), and by an Intern Clinical Psychologist (TL) with a PhD qualification in psychology.^2^

*The Beck Depression Inventory – Second Edition* (BDI-II, Beck, et al., 1996, alpha = .83) and *Beck Anxiety Inventory* (BAI, Beck & Steer, 1996, alpha = .81) assessed the severity of depressive and anxiety symptoms, respectively, over the past fortnight.

*The Intrusive Memory Interview* (IMI, following Hackmann, Ehlers, Speckens, & Clark, 2004) was used as a self-report measure of participants' subjective experience of a negative intrusive memory in the preceding week. Participants read a definition of intrusive memories,^3^ and then described the content of one memory that was most distressing and intrusive. Participants recorded their age at the event in the memory, and the time that had passed since the event. Anchoring responses to the past week, they rated the degree of intrusion-related distress they had experienced on a 0 (*not at all*) to 100 scale (*very much*) scale. See Table 1 for memory content.

The 15-item *Impact of Event Scale* (IES, Horowitz, et al., 1979) assessed the degree of intrusion (e.g., “I thought about it when I didn't mean to”) and avoidance (e.g., “I tried not to think about it”) of participants' intrusive memory. Participants rated each statement on a 4 point scale over the past week, where 0 = *not at all*, 1 = *rarely*, 3 = *sometimes*, and 5 = *often*. Internal reliabilities were .73 (Intrusion), .72 (Avoidance).

The *Appraisals of Intrusive Memories Questionnaire* (see Newby & Moulds, 2010 for a complete list of items) is a self-report measure of appraisals. Participants rated the degree to which they believed 25 appraisals (e.g., “Having this memory means I will lose control”, “Having this memory means I have a psychological problem”) at the time when they had experienced their intrusive memory in the preceding week (from 0 to 100, where 0 = *did not believe this*, 100 = *completely convinced*). Items were averaged to derive a negative appraisals of intrusive memories score (alpha = .92).

The *Positive and Negative Affective Scale* (PANAS, Watson, Clark, & Tellegen, 1988) measured positive (10 items e.g., ‘enthusiastic’, alpha = .82) and negative affect (10 items e.g., ‘upset’, alpha = .78) on a 1 (*very slightly or not at all*) to 5 (*extremely*) scale in the present moment both before [PANAS (pre)] and after [PANAS (post)] the intervention.

*Intrusive Memory Diary* (Lang et al., 2009). Participants kept a daily diary of any occurrence of their intrusive memory for seven days, and were instructed to fill out the diary at least once per day, regardless of whether they had experienced an intrusion.

*Expectancy ratings, diary compliance ratings, and ratings of acceptability of the interventions.* Immediately following training, to measure expectancy, participants in the CBM and CB-Education groups were asked: “At this point how helpful do you think the material covered in the session will be for you?” on a 0 (*not at all helpful*) to 10 (*extremely helpful*) scale.^4^ To assess compliance with completion of the diary, all participants rated how accurate they thought their diary was on a 0 (*not at all accurate*) to 10 (*extremely accurate*) scale. After participating, all participants rated how enjoyable the study was, the extent to which they benefited from participating, the acceptability of the procedures in helping them with their memories, and how confident they felt in being able to cope with other intrusive memories, on 0 (*not at all/did not benefit*) to 10 (*very enjoyable, I benefited a lot, very acceptable, very confident*) scales.

#### Materials for the CBM session

The *CBM task* (positive CBM, Lang et al., 2009, based on Mathews & Mackintosh, 2000) was used to train positive appraisals of intrusive memories. The training stimuli consisted of 72 intrusive memory appraisal-related sentences (scripts) that were positively valenced (e.g., “Intrusive memories mean nothing is wrong with me”, “Having intrusive memories mean that I can cope”) in addition to eight neutral fillers (e.g., “Intrusive memories pop into my mind spontaneously”). Participants viewed and processed each script in the form of a sentence completion task. For example, for the script “Having intrusive memories means that nothing is wrong with me”, participants were presented with the first half of the script (e.g., “Having intrusive memories means that”) for 2 s, and then the final half of the statement was presented in the form of a word fragment (e.g., “n – thing is wrong w – th me”). The participant's task was to complete the word fragment by typing on the keyboard the first missing letter (i.e., ‘o’). The correct words then appeared on the screen (i.e., “nothing is wrong with me”), regardless of whether the participant completed the word fragment correctly. To encourage participants to process the meaning of the sentences, a comprehension question that reinforced the positive interpretation was included after half of the sentences. Error feedback for incorrect responses was provided. For instance, the comprehension question for the example above was “Do you believe intrusive memories mean something is wrong with you?” If participants indicated ‘yes’, they saw the word ‘incorrect’ in red on the computer screen. Questions were designed such that “yes” and “no” answers occurred equally often. After being provided with task instructions,^5^ participants completed two practice trials. Following the practice trials, participants were given the opportunity to ask questions before they commenced the experimental items.

#### Materials for the CB-Education session (available from first author)

The CB-Education session included psychoeducation, cognitive challenging and two behavioural experiments. *Psychoeducation.* Following a standardised script, the experimenter normalised the experience of intrusions, explained the link between negative appraisals of intrusive memories and distress/negative emotions, and then provided a rationale for conducting cognitive challenging and experiments to test the validity of their beliefs about intrusive memories. During *Cognitive Challenging*, the experimenter challenged a negative appraisal (either “Having this memory means I am inadequate” or “Having this memory means I am inferior”), by asking participants a series of verbal questions (e.g., identifying the advantages/disadvantages of the appraisal). Participants were prompted to write their responses to each question on paper. The two *Behavioural Experiments* followed the structure outlined in Bennett-Levy et al. (2004). Experiment 1 was designed to test the validity of the target appraisal “I should be able to rid my mind of this memory completely.” The experiment used an adaptation of Wegner's thought suppression paradigm (Wegner, Schneider, Carter, & White, 1987) to demonstrate the futility of thought/memory suppression. First, the experimenter provided a brief introduction ‘*There are two experiments we will do today to test some beliefs that some people hold about their memories which make them upset when they experience their memories. The first one is that some people believe that they should be able to control their memories and get rid of them from their minds completely.’* Second, the experimenter asked participants to rate the strength of their belief (from 0 to 100) in this target appraisal. Third, participants were informed that they would soon be asked to suppress their thoughts about chocolate cake for one minute (as an analogue to assess their ability to suppress their intrusive memory), and were prompted to write down their predictions about their ability to suppress their thoughts. Fourth, participants then attempted the thought suppression task, and finally, evaluated the outcome of the task with the experimenter (e.g., “What did you learn about your ability to suppress thoughts?” “Do you still think that you are able to rid the memory from your mind altogether?” What is your rating of the target appraisal now from 0 to 100?).

Experiment 2 was designed to test the validity of the appraisal “This memory will make me feel upset for a long time and will interfere with a task that I am trying to complete.” First, participants rated the strength of their belief in this appraisal (from 0 to 100). Next, they were informed by the experimenter that they would be asked to describe their intrusive memory out loud, and then complete a short written task. Prior to describing their intrusive memory, participants were prompted to write down their predictions, rated the amount of distress they expected (0 to 100 scale), and also rated the degree to which they expected the recall of the memory would interfere with their ability to complete the written task. Next participants completed the task. Finally, the experimenter reviewed the outcome of the experiment with the participant (in relation to the target appraisal).

### Procedure

*Day 1 (baseline, at home)*. Participants completed a questionnaire package (that included an information statement and consent form, demographics questionnaire, BDI-II, BAI, IMI, IES and negative appraisals questionnaire) on Day 1 at home, and brought the completed questionnaires with them to the laboratory on Day 2.^6^

*Day 2 (laboratory)*. Participants were administered the SCID-I. Next, they completed the PANAS (pre). Following this, the three experimental groups had different procedures. The Control group was instructed how to complete the memory diary and then arranged the follow-up session with the experimenter. Participants in the CBM and CB-Education conditions completed the CBM task (or CB-Education components), followed by the PANAS (post) and expectancy rating, and were finally provided with diary completion instructions before arranging the follow-up session.

*Days 2–8 (at home)*. Participants completed the intrusive memory diary each day at home.

*Day 9 (one week follow-up, laboratory)*. Participants returned to the laboratory and completed a shortened version of the IMI (that excluded questions about content, time since event, age at event), and completed the IES, appraisals questionnaire, BAI and an adapted version of the BDI-II (mood over the past week, rather than fortnight). All measures were anchored to their experience of their intrusive memory (or mood/anxiety) over the previous week between baseline and follow-up. Finally, participants completed their diary compliance and acceptability ratings.

## Results

### Participant demographics and baseline between-group comparisons

Participants were on average 26 years, and the majority were Asian or Caucasian, unmarried, university educated, and unemployed. The sample consisted of primarily clinically depressed individuals with over 88 percent of the sample meeting criteria for either major depressive disorder (MDD; current or past) or dysthymic disorder (see Table 2). Chi square analyses and one-way analyses of variance (ANOVAs) confirmed that the groups were matched on demographic characteristics, diagnostic status, intrusive memory variables, mood and anxiety (*F*s < 1, *p*'s > .05) (see Table 2 for demographics).

### Expectancy ratings (CB-Education and CBM)

Independent samples *t*-tests showed that there were no significant differences between the CBM and CB-Education groups on expectancy ratings of how helpful they thought the interventions would be (*t* (33) = .04, *p* = .96)^7^ (Table 4).

### Effects of training on state mood

To explore the impact of the active interventions on state mood, analyses of covariance (ANCOVA) were conducted comparing PANAS (post) mood (positive/negative) scores between the active training groups (CB-Education vs. CBM), with PANAS (pre) mood (positive/negative) scores entered as a covariate. There were no significant differences following the training session (PANAS (post) positive: *F* (2, 35) = 0.0, *p* = .97; PANAS (post) negative scores: *F*(2, 35) = 0.52, *p* = .48).

### One-week follow-up data

Mixed model ANOVAs were conducted with a between-subjects factor of experimental condition (3: CBM vs. CB-Education, vs. Control) and a within-subjects factor of time (2: baseline vs. one-week follow-up) on mood and intrusion variables to assess for reductions over time, and group differences at one-week follow-up.

#### Depression and anxiety, intrusion and avoidance (IES scores), and negative appraisals

We found main effects of time for BDI-II, BAI, IES Intrusion, and negative appraisals scores (*F*'s > 8.8, *p*'s < .01), but not IES Avoidance (*p* = .14). The time by condition interactions were not significant for BDI-II,^8^ BAI, IES Intrusion nor IES Avoidance (*p*'s > .05). Means and statistics are presented in Table 3.

### Intrusion-related distress

We found a main effect of time for intrusion-related distress, as well as a time by condition interaction (*ps* < .001). To decompose this interaction, we calculated the mean change in intrusion-related distress scores between baseline and follow-up for each group (distress change scores, Control: *M* = 9.00, *SD* = 16.19; CBM: *M* = 19.50, *SD* = 23.89; CB-Education: *M* = 28.50, *SD* = 27.39). Independent samples *t*-tests demonstrated that the CB-Education group reported greater reductions in intrusion-related distress compared to the Control group (*t*(38) = 2.74, *p* < .01, Cohen's *d* = .89). In contrast, there were no significant differences in distress change scores between the CBM and CB-Education groups (*t*(38) = 1.11, *p* = .27, Cohen's *d* = .35), nor between the Control and CBM groups (*t*(38) = 1.63, *p* = .11, Cohen's *d* = .52).

### Intrusive Memory Diary

There were no between-group differences on the frequency of intrusive memories recorded in the daily diary (*F* (2, 56) = 2.30, *p* = .11, see Table 4). Participants reported moderate levels of accuracy in their completion of the diary (*M* = 7.81, *SD* = 1.06) with no significant between-group differences (*F* (2, 57) = 1.55, *p* = .21).

### Correlational analyses

To explore whether the changes in negative appraisals were associated with changes in distress scores, we carried out a Pearson's *r* correlation between negative appraisals change scores (i.e., between baseline to follow-up) and distress change scores. This association was significant (*r* = .33, *p* < .01), indicating the larger reduction in negative appraisals between baseline and follow-up was associated with larger reduction in intrusion-distress.

### Acceptability of procedures ratings

Importantly, there were no between-condition differences in participants' ratings of acceptability of the procedures (enjoyment: *F* (2, 57) = 1.40, *p* = .25, benefit: *F* (2, 57) = .05, *p* = .95, acceptability: *F* (2, 57) = .26, *p* = .77, nor confidence in coping with other intrusive memories: *F* (2, 57) = 2.52, *p* = .089) (Table 4). The ratings were on average, moderate to high.

## Discussion

This study sought to evaluate and compare two interventions (a session of CBM positive appraisal training versus one session of therapist-delivered CB-Education) that both aimed to alter maladaptive appraisals of a negative intrusive autobiographical memory and associated distress. A sample of dysphoric participants (over 88% of whom met diagnostic criteria for a DSM-IV diagnosis of MDD or Dysthymia), who were primarily experiencing imagery-based intrusions of negative interpersonal events, were allocated to CBM, CB-Education or Control conditions and were followed up one week later. For all groups, there were significant reductions over one week in mood (depression and anxiety), memory intrusiveness and negative appraisals.

All three groups reported that their intrusive memory was significantly less distressing at one-week follow-up, with the CB-Education group reporting the greatest reduction in distress, followed by the CBM group. This result cannot be explained by differences in the emotional impact of training on state mood, or expectation factors (see MacLeod, Koster, & Fox, 2009), but we cannot rule out the possibility that demand factors influenced the CB-Education group. However, if demand was the sole cause of group differences in distress ratings at one week, we would expect to have seen the same pattern of results for negative appraisal ratings, but this was not the case. The results suggest that intrusion-related distress can be reduced using CB-Education and CBM interventions that target negative appraisals of intrusions. In addition, CB-Education (and potentially, CBM) is more efficacious at reducing distress than assessment and self-monitoring of intrusive memories in dysphoric individuals. Finally, our correlational analysis suggests that reductions in negative appraisals were associated with reductions in intrusion-related distress. Future research is now needed to replicate the findings in larger samples and establish the causal mechanism that drove the reductions in distress.

The finding that memory intrusiveness (IES Intrusion scores), negative appraisals and mood symptoms (depression and anxiety) reduced in all three groups was unexpected. This finding raises the possibility that the shared components that formed part of all three conditions (self-report measures, SCID-I/NP assessments and daily memory diary) facilitated these reductions. Completion of the self-report measures (or the diary) may have normalised the experience of intrusive memories, inadvertently challenging any maladaptive beliefs, or facilitated some exposure to and/or emotional processing of the memory. While these results highlight the potentially powerful role of assessment and monitoring, we acknowledge that they are not sufficient as a standalone intervention. Future studies that involve multiple baseline assessments to ensure the stability of symptoms prior to taking part in the interventions, and the adoption of a control condition that is limited to brief assessment (without monitoring), would further clarify the potential benefit of assessment.

### Limitations

This study may not have had sufficient power to detect group differences between the CBM and CB-Education groups because of small sample sizes. For example, although the effect size for the between-group comparison between CB-Education versus CBM groups on intrusion-related distress suggest a small group difference (*d* = 0.35), this difference did not reach statistical significance. This was likely due to the study being underpowered to detect small group differences. Therefore, given the lack of power, we cannot conclude that CB-Education and CBM were equivalent, nor whether one group was superior to the other. Replication of this study is now needed with larger samples to compare the efficacy of CB-Education and the CBM training on intrusive memories and associated variables.

This study also awaits replication in a sample of individuals with MDD. In addition, our results are solely reliant upon self-report measures. Further investigations are needed to evaluate whether the findings are observed using objective physiological measures (e.g., skin conductance). There was large variability in participant responses on most measures of intrusions (including appraisals) at baseline. It is possible that CBM and CB-Education are more useful when delivered to individuals who have strong or resistant cognitive biases related to intrusions. Moreover, the interventions were limited to a single session and short follow-up. Further investigations are needed with longer-follow ups to explore the maintenance of gains, and whether the efficacy of the active interventions is enhanced with repeated administration. Clarification of these issues will inform us of how CBM can be most effectively used when translated to clinical settings. Finally, the CB-Education and CBM interventions were not matched on a number of important variables that may have confounded our results (e.g., therapist contact, inclusion of a rationale prior to training, the specificity of the targeted appraisals).

## Conclusions

Despite these limitations, this study provided a novel comparison between two methods (computerised training versus therapist delivered intervention) that aimed to target negative appraisals of intrusive (image based) memories in a dysphoric sample. This study has extended previous work by investigating the utility of CB-Education and CBM in targeting a distressing intrusive autobiographical memory rather than a memory of a laboratory-based stressor, and importantly, by including a control condition. Our results cautiously suggest the promise of using both CB-Education session and CBM techniques to reduce intrusion-related distress, but this study needs replication with longer follow-ups, repeated administration, and larger samples. Finally, our findings underscore the importance of thorough assessment of intrusive memories in treatments for depression, which appears to be potentially beneficial in reducing negative intrusive memories and maladaptive negative appraisals of these memories in dysphoria.

## Figures and Tables

**Table 1:** Content, frequency and age of negative intrusive memories at baseline.

	CBM *n* = 20	CB-Education *n* = 20	Control *n* = 20	Statistic
Content				
Interpersonal event	12 (70.6)	13 (76.5)	11 (64.7)	
Death/illness involving other	1 (5.9)	1 (5.9)	2 (11.8)	
Personal Assault/abuse	0 (0)	1 (5.9)	0 (0)	
Illness/injury involving self	0 (0)	1 (5.9)	0 (0)	
Failure (uni/work)	2 (11.8)	1 (5.9)	3 (17.7)	
Other	2 (11.8)	0 (0)	1 (5.9)	
Frequency during day				
1–2 times	7 (41.2)	9 (52.9)	9 (52.9)	
3–5 times	9 (52.9)	8 (47.1)	6 (35.3)	
5–10 times	1 (5.9)	0 (0)	2 (11.8)	
Frequency past week_a_	4.59 (1.54)	4.00 (1.87)	3.76 (1.98)	*F* (2, 48) = .93, *p* = .40
Time since event (weeks)_a_	143.59 (129.42)	154.24 (192.10)	91.59 (142.36)	*F* (2, 48) = .77, *p* = .47
Age at event_a_	26.00 (11.93)	22.88 (12.24)	23.76 (8.20)	*F* (2, 48) = .37, *p* = .69

*Note.* Except where noted, values refer to frequency (percentage) scores. a = Values refer to mean and (standard deviation) scores. CBM = Computerised Bias Modification, CB-Education = Cognitive Behavioural Education.

**Table 2:** Sample and demographic characteristics.

	CBM *n* = 20	CB-Education *n* = 20	Control *n* = 20	Statistic
Gender *n* (% female)_a_	16 (80.0)	16 (80.0)	14 (70.0)	*χ*^2^ (2, *N* = 60) = .74, *p* = .69
Age (years)	28.05 (12.39)	25.30 (10.16)	25.50 (7.01)	*F* (2, 57) = .46, *p* = .63
Marital Status_a_				
Single	16 (80.0)	16 (80.0)	14 (70.0)	Single: *χ*^2^ (2, *N* = 60) = .74, *p* = .69
Married	1 (5.0)	3 (15.0)	4 (20.0)	
De-Facto	2 (10.0)	1 (5.0)	1 (5.0)	
Separated/Divorced	1 (5.0)	0	1 (5.0)	
Other	0	0	0	
Educational History_a_				
Completed year 12	7 (35.0)	0 (50.0)	7 (35.0)	Bachelor: *χ*^2^ (2, *N* = 60) = .40, *p* = .82
Completed Bachelor	9 (45.0)	8 (40.0)	10 (50.0)	
Completed Masters	1 (5.0)	1 (5.0)	3 (15.0)	
Completed PhD	1 (5.0)	0	0	
Other	2 (10.0)	1 (5.0)	0	
Employment Status_a_				
Unemployed	11 (55.0)	14 (70.0)	12 (60.0)	Unemployed: *χ*^2^ (2, *N* = 60) = .99, *p* = .61
Employed (Casual/Part-Time)	8 (40.0)	5 (25.0)	6 (30.0)	
Employed Full-Time	1 (5.0)	1 (5.0)	2 (10.0)	
Ethnicity_a_				Asian ethnicity: *χ*^2^ (2, *N* = 60) = 1.62, *p* = .45
Caucasian	8 (40.0)	8 (40.0)	6 (30.0)	
Asian	11 (55.0)	9 (45.0)	13 (65.0)	
Indian/Pakistani	1 (5.0)	3 (15.0)	1 (5.0)	
Current episode (months)	11.56 (15.45)	27.58 (35.76)	9.95 (14.96)	*F* (2, 28) = 1.64, *p* = .21
Number of previous episodes				
First episode	3 (17.6)	4 (23.5)	2 (11.8)	
One to two episodes	5 (29.4)	6 (35.3)	10 (58.8)	
Three or more	6 (35.3)	5 (29.4)	2 (11.8)	
Depression Status_a_				MDD: *χ*^2^ (2, *N* = 60) = 1.43, *p* = .49
Recovered MDD	5 (25.0)	2 (10.0)	2 (10.0)	
Current MDD	12 (60.0)	15 (75.0)	15 (75.0)	
Dysthymic Disorder	1 (5.0)	1 (5.0)	0 (0)	
Subthreshold MDD	0 (0)	1 (5.0)	1 (5.0)	
No diagnosis	2 (10.0)	1 (5.0)	2 (10.0)	
Current Medications_a_	4 (20.0)	5 (25.0)	5 (25.0)	*χ*^2^ (2, *N* = 60) = .19, *p* = .91
Current Treatment_a_	5 (23.8)	4 (20.0)	5 (25.0)	*χ*^2^ (2, *N* = 60) = .19, *p* = .91
Previous Treatment_a_	9 (45.0)	8 (40.0)	8 (40.0)	*χ*^2^ (2, *N* = 60) = .14, *p* = .93

*Note.* a = Values refer to frequency (percentage) scores. Except where otherwise noted, values refer to mean and (standard deviation) scores. CBM = Computerised Bias Modification, CB-Education = Cognitive Behavioural Education. MDD = Major Depressive Disorder. Recovered MDD refers to people who meet criteria for a past history of Major Depressive Disorder, but do not currently meet criteria for MDD.

**Table 3:** Mood, anxiety, IES scores, negative appraisals and avoidance results (baseline and follow-up).

	CBM				CB-Education				Control				Main effect of Time	Time × Condition interaction
	Baseline		One week follow-up		Baseline		One week follow-up		Baselin		One week follow-up			
	*M*	*SD*	*M*	*SD*	*M*	*SD*	*M*	*SD*	*M*	*SD*	*M*	*SD*		
BDI-II	26.70	9.55	20.65	9.44	30.16	8.27	24.05	12.44	26.74	8.17	25.21	10.55	*F* (1, 48) = 11.29, *p* < .01, partial eta-squared = .20	*F*(2,46) = 1.28, *p* = .29, partial eta-squared = .05
BAI	20.95	8.18	14.50	7.98	20.95	10.55	16.30	11.08	21.75	13.03	20.30	13.23	*F* (1,57) = 15.61, *p* < .001, partial eta-squared = .21	*F* (2,57) = 1.91, *p* = .16, partial eta-squared = .06
IES Intrusion	20.00	7.70	17.35	8.53	21.80	7.67	16.75	7.28	22.90	7.70	21.35	7.23	*F* (1, 57) = 11.16, *p* < .001, partial eta-squared = .16	*F*(2, 57) = 1.25, *p* = .29, partial eta squared = .04
IES Avoidance	18.80	10.25	18.90	8.25	21.80	8.67	17.55	6.57	20.10	8.21	20.25	7.27	*F* (1, 57) = 2.21, *p* = .14, partial eta-squared = .04	*F* (2, 57) = 2.65, *p* = .080, partial eta-squared = .09
Negative Appraisals	30.67	17.40	24.08	18.82	31.72	17.61	22.74	14.74	36.08	20.85	31.58	22.05	*F* (1,57) = 8.83, *p* < .01, partial eta-squared = .13	*F* (2,57) = .33, *p* = .72, partial eta-squared = .01
Distress	77.00	20.03	57.5	19.23	70.50	22.12	42.00	22.80	73.00	20.80	64.00	22.80	*F* (1, 57) = 41.04, *p* < .001, partial eta-squared = .42	*F* (2, 57) = 3.61, *p* = .03, partial eta-squared = .11

*Note.* BDI-II = Beck Depression Inventory, BAI = Beck Anxiety Inventory, IES = Impact of Event Scale, *M* = mean, *SD* = standard deviation. CBM = Computerised Bias Modification, CB-Education = Cognitive Behavioural Education.

**Table 4:** PANAS ratings, diary frequency, diary compliance and acceptability ratings.

	CBM *n* = 20 *M* (*SD*)	CB-Education *n* = 20 *M* (*SD*)	Control *n* = 20 *M* (*SD*)	Statistic
PANAS				
PANAS positive (pre)	18.90 (6.08)	16.21 (4.25)	26.18 (7.63)	*t*(37) = 1.59, *p* = .12
PANAS positive (post)	20.63 (8.23)	18.26 (6.03)	–	*t*(36) = 1.01, *p* = .32
PANAS negative (pre)	20.50 (6.58)	19.53 (6.97)	24.35 (7.87)	*t*(37) = .45, *p* = .65
PANAS negative (post)	15.68 (5.61)	16.42 (7.04)	–	*t*(36) = −.36, *p* = .72
Expectancy rating	6.59 (2.24)	6.56 (1.92)	–	*t*(27) = .04, *p* = .96
Diary				
Diary frequency	8.50 (7.05)	10.15 (6.72)	14.47 (12.19)	*F* (2, 58) = 2.98, *p* = .059
Diary accuracy	8.13 (1.12)	7.75 (.91)	7.55 (1.10)	*F* (2, 57) = 1.55, *p* = .21
Acceptability ratings				
Enjoyment from participation	6.55 (2.11)	5.60 (2.23)	6.55 (1.85)	*F* (2, 57) = 1.40, *p* = .25
Benefit from participation	6.90 (2.00)	6.70 (2.05)	6.85 (2.11)	*F* (2, 57) = .05, *p* = .95
Acceptability of Procedure	7.20 (2.21)	6.75 (2.00)	7.05 (1.73)	*F* (2, 57) = .26, *p* = .77
Confidence in coping with IMs in future	6.80 (2.02)	5.80 (1.73)	5.35 (2.45)	*F* (2, 57) = 2.52, *p* = .09

*Note.* CBM = Computerised Bias Modification, CB-Education = Cognitive Behavioural Education, PANAS = Positive and Negative Affect Schedule. For the CBM and CB-Education conditions, the PANAS was administered both before (pre) and after (post) the intervention. Items were summed to derive PANAS scores. The Control group completed the PANAS (pre) measure to ensure that the groups were matched at baseline. IMs = intrusive memories. Due to missing data, there were only 17 and 18 participants in the CBM and CB-Education groups respectively who completed the expectancy rating.

## Footnotes

1 This project was approved by the Human Research Ethics Advisory Panel at the University of New South Wales. All participants provided written informed consent prior to participation.

2 To determine inter-rater reliability, 9 (17.4%) of the participants were re-interviewed by another Clinical Psychologist within two weeks of initial interview. With the exception of a discrepant diagnosis recorded for one participant, there was complete agreement between interviewers in their diagnostic assessments.

3 “Sometimes we remember things without trying to. We have memories that just pop into our mind spontaneously or when we do not want them to. For example, if you had an argument with a friend awhile ago and you recalled memories about this argument such as recalling what occurred or remembering what was said, when you were not deliberately thinking about the argument, we would call these spontaneous memories. Spontaneous memories can occur based on any past event that you have actually experienced. They might be triggered by reminders of the event, but these memories are different than general thoughts or worries about things because they are memories of specific events or incidents which have actually happened to you.”

4 We did not include this item in the Control group because we wanted to minimise the possibility of inducing demand factors that could have resulted from including this question in the Control materials.

5 Participants were instructed to read a series of statements about intrusive memories and were instructed about how to complete the task. No other instructions were provided, and no rationale for the task was provided.

6 Participants were asked to complete the questionnaires at home (as opposed to in the laboratory with the experimenter present) because we wanted to reduce the likelihood of normalising the experience of intrusive memories in the face-to-face session.

7 Due to missing data, there were only 17 and 18 participants in the CBM and CB-Education groups respectively who completed the expectancy rating.

8 Baseline BDI-II data was missing for one CB-Education participant and follow-up BDI-II data was missing for a second CB-Education participant.
